# Editorial: Golgi Dynamics in Physiological and Pathological Conditions

**DOI:** 10.3389/fcell.2020.00007

**Published:** 2020-01-29

**Authors:** Kristian Prydz, Vladimir Lupashin, Yanzhuang Wang, Jaakko Saraste

**Affiliations:** ^1^Department of Biosciences, University of Oslo, Oslo, Norway; ^2^Department of Physiology and Biophysics, University of Arkansas for Medical Sciences, Little Rock, AR, United States; ^3^Department of Molecular, Cellular and Developmental Biology, University of Michigan, Ann Arbor, MI, United States; ^4^Department of Biomedicine and Molecular Imaging Center, University of Bergen, Bergen, Norway

**Keywords:** Golgi apparatus, Golgi dynamics, Golgi structure, Golgi function, membrane trafficking

The internal reticular apparatus, first reported in 1898, and later called the Golgi apparatus, was initially observed by Camillo Golgi after his refinement of the silver nitrate technique for staining cells in the nervous system, for which he received the Nobel Prize in Physiology or Medicine in 1906. The true existence of the Golgi apparatus was, however, disputed for decades, and many scientists regarded it as a staining artifact (Palade and Claude, 1949). Only after the electron microscope became a more generally available tool for cell biologists was the Golgi apparatus accepted as an authentic cellular structure, and its existence demonstrated in practically all eukaryotic cells, from yeast to man. However, for reasons that are not completely understood, the Golgi assumes various forms in different cell types, ranging from tubular networks or individual *cisternae* (budding yeast *S. cerevisiae*) to separate cisternal stacks (e.g., invertebrates and plants) and a ribbon-like structure of interconnected stacks (vertebrates). As reflected in this collection, much of the recent efforts to define the mechanism of Golgi structure formation have been put on Golgi structural proteins including Golgins and GRASPs, actin and microtubule cytoskeletons, Rabs, and other GTPases, as well as certain other proteins (Ahat et al.; Egorov and Polishchuk; Lowe; Phuyal and Farhan; Satoh et al.; Shaik et al.). Although these proteins have their own characteristic functions, they coordinate with each other to maintain the structural and functional integrity of the Golgi.

The fascinating structure of the Golgi stacks is partly determined by GRASP proteins which also participate in a number of additional cellular processes, such as unconventional secretion and autophagy (Ahat et al.). In vertebrate cells, the multiple stacks are further organized into the Golgi ribbon, a continuous structure where the cisternal stacks are joined together by lateral connections (Saraste and Prydz). Several proteins have been shown to be involved in the regulation of ribbon structure, among these GRASP55 and GRASP65 (Ahat et al.) and the tethering protein giantin (Satoh et al.). Although the ribbon-like organization of the Golgi remains enigmatic, it is highly interesting with respect to certain pathological situations. Indeed, fragmentation of the Golgi ribbon is encountered in a number of neurological diseases (Caracci et al.; Makhoul et al.). The consequences of Golgi structural abnormalities on its function and cellular activities may vary in different experimental or disease conditions. For example, knocking down the GRASP proteins accelerates intra-Golgi trafficking (Xiang et al., 2013; Lee et al., 2014), while inhibition of GM130 and p115 results in the accumulation of COPI vesicles and reduced membrane trafficking (Seemann et al., 2000). Similarly, Aβ (amyloid β)-induced Golgi reorganization in Alzheimer's disease has been proposed to increase APP (amyloid precursor protein) trafficking and Aβ production (Joshi et al., 2014), while GM130 knockout slows down ER-Golgi trafficking, resulting in Purkinje neuron loss and ataxia in mice (Liu et al., 2017). In addition, both the morphology (Makhoul et al.) and the internal environment (such as lumenal pH) of the Golgi apparatus are altered in cancer cells, leading to changes in glycosylation (Kellokumpu). Future investigations of the cause and effect of Golgi defects in disease will undoubtedly yield exciting findings essential for the understanding of both Golgi function and disease development.

Structural reorganization of the Golgi is also an integral part of naturally occurring processes, as best demonstrated in the case of cell division. During the late G2 phase, as cells prepare for mitosis, the Golgi ribbon is unlinked into individual stacks, which subsequently undergo further disassembly and vesiculation. In the course of these events, repositioning of Golgi elements is typically observed in the perinuclear region of many vertebrate cells, controlled by the duplicated centrosome and centrosome-nucleated microtubules of the forming mitotic spindle (Mascanzoni et al.). Notably, the pulling apart of the centrosomes during the late G2/early mitosis is accompanied by the evidently equal partitioning of the intermediate compartment (IC), a permanent membrane network that–unlike the Golgi stacks—keeps its properties during mitosis (Saraste and Marie, 2018).

The role of the Golgi apparatus as an important way station in anterograde trafficking along the secretory pathway was established during the 1960s and 1970s (see Farquhar and Palade, 1998, for a review), while retrograde transport via this organelle was described much later (Sandvig et al., 1992). In addition, the role of the Golgi apparatus as an “educational” site for glycoproteins, proteoglycans, and glycolipids is well-described and generally accepted. Accordingly, the majority of newly synthesized proteins that enter the Golgi apparatus at its *cis*-side carry N-linked glycans of identical structure, but leave the *trans*-Golgi region equipped with highly diverse glycans, specific for the actual species, the cell type, as well as the cell's developmental stage or degree of differentiation (Fisher et al.; Akintayo and Stanley). The ability of various transiting cargo molecules to obtain a healthy output of these and other Golgi modifications, however, requires mechanisms of membrane homeostasis and transport that are still subject to active investigation and dispute (Mironov and Beznoussenko; Saraste and Prydz; Makhoul et al.; Pantazopoulou and Glick). Not only the evaluation of competing Golgi models (Mironov and Beznoussenko), but also performing mathematical modeling (Fisher et al.) can advance our understanding of how cargo molecules that enter the *cis*-face of the Golgi apparatus are modified during Golgi transit, and how their final structures will turn out. The ongoing attempts to correlate the structural and functional dynamics of the Golgi apparatus are still absolutely required to achieve this goal. While the mechanisms of how different cargo molecules traffic through the Golgi stacks are still under debate, it remains even less clear how precise localization of Golgi resident proteins is achieved within the polarized stacks.

The swarms of COPI vesicles observed at the outskirts of the Golgi membranes are essential for normal Golgi function, although their engagement is still not fully understood due to partial knowledge of their exact cargo selection and composition, places of origin and destination, delivery mechanisms, interaction partners and regulatory modes (Pantazopoulou and Glick; Luo and Boyce; D'Souza et al.). The Rho GTPase Cdc42 is involved in the regulation of actin filament- and microtubule-dependent Golgi positioning, in addition to interacting with COPI vesicles or tubules, thus potentially promoting anterograde transport toward the leading edge of migrating cells (Phuyal and Farhan). The trafficking capacity of these transport intermediates can be regulated in a number of ways (Luo and Boyce). For example, defects in the octameric COG complex that functions as a tether in COPI vesicle-mediated retrograde transport, not only affect traditional Golgi functions like glycosylation and sorting, but also exert effects elsewhere in the cell, in particular within the endo-lysosomal system (D'Souza et al.). Knockdown of the COG3 subunit of COG–or the ZW10 subunit of NRZ/Dsl1, another member of the CATCHR family of multisubunit tethering complexes–leads to the dispersal of the Golgi apparatus throughout the cytoplasm of metazoan cells (Zolov and Lupashin, 2005; Sun et al., 2007) in a process that requires both Rab GTPases and kinesin motor proteins (Liu et al.). The dynamic nature of the Golgi apparatus is underlined by the fact that while a large number of proteins are required to maintain its normal organization, treatments affecting a single structural or machinery component are often sufficient to destabilize its structure, as exemplified by the EGFR tyrosine kinase inhibitors BML-265 and AG1478 (Boncompain et al.).

In addition to the COPI transport machineries, the dynamic nature and maintenance of the Golgi apparatus crucially depend on efficient mechanisms of membrane fusion and fission. Both processes are influenced by lipid modifying enzymes, such as acylglycerophosphate acyltransferases (Zhukovsky et al.), membrane curvature-sensing proteins, and fission inducing-proteins (Zhukovsky et al.). To understand Golgi function completely it will be important to reconstitute Golgi fusion and fission *in vitro* using purified components and endogenous cargo.

A question that has been touched upon, but is far from being solved, is whether all the stacks in a Golgi ribbon handle the same cargo and have identical enzymatic contents to carry out the same protein and lipid modifications. While the non-linked, wide-spread Golgi stacks in *Drosophila* cells differ in their enzymatic repertoire (Yano et al., 2005), the apical and basolateral routes in mammalian epithelial MDCK cells have also been shown to treat the same cargo molecule differently (Prydz et al., 2008). A related question is whether there may be lateral segregation of cargo within *cisternae* of the same Golgi stack. Interestingly, this was recently shown to be the case for HeLa cells, where two proteins heading for the endo-lysosomal system gradually underwent lateral segregation while passing through the Golgi (Chen et al., 2017). Here, the paper by Ernst et al. discusses recent findings showing that acylation can influence the lateral positioning of proteins in Golgi cisternae, and as a consequence, their anterograde transport efficiency (Ernst et al.). While anterograde transport of cargo also takes place after the stacks of the Golgi ribbon have been dispersed, for instance, due to breakdown of microtubules, an intact ribbon does seem to be important for the transport of large cargo molecules (Lavieu et al., 2014). In a new view of the Golgi ribbon, the non-compact zones linking the cisternal stacks are jointly occupied by the permanent IC elements and recycling endosomes (RE). As mentioned above, as a prelude for cell division, the stacks that undergo reversible break-down disconnect from these centrosome-linked compartments, which as a consequence would be allowed move to the spindle poles for partitioning into the forming daughter cells (Saraste and Marie, 2018; Saraste and Prydz).

Indeed, the Golgi apparatus is in intimate communication with both pre- and post-Golgi compartments. A number of important proteins recycle between the endoplasmic reticulum (ER) and the Golgi apparatus. YIPFα1A, a member of the YIPF protein family, functions at ER exit sites and interacts with COPII components, but can also localize to the IC and *cis*-Golgi, interact with YIPβ1A, and recycle back to the ER (Shaik et al.). Members of the CREB3 family of transcription factors move from the ER to the Golgi apparatus when the cell receives a proper signal. The activation of these proteins in the Golgi apparatus is based on two sequential proteolytic cleavage events. Upon cleavage, the N-terminal portions of the proteins, which are localized to the cytoplasmic side of the Golgi membrane, are released and become free to move into the nucleus (Sampieri et al.).

At the *trans*-side of the Golgi stacks, the *trans*-Golgi network (TGN) is an important site for Golgi exit of cargo molecules destined for various organelles of the endomembrane system and different plasma membrane domains. With the continuous improvement of the resolution of fluorescence-based light microscopes, it is now possible to observe the segregation of various cargo molecules and the membrane carriers that exit the Golgi at the level of the TGN (Huang et al.).

To fully (or at least better) understand the Golgi apparatus in various physiological and pathological conditions, one has to examine this organelle in a variety of tissues at different stages of differentiation, development or degeneration, for instance in neuronal (Caracci et al.; Rabouille and Haase, 2016) and muscle cells (Oddoux et al.). This is important to understand the adaptability of the Golgi apparatus to the cellular requirements, ensuring a healthy glycan output, as exemplified in this collection by the analysis of glycans of oocytes and sperm cells (Akintayo and Stanley). Equally important is to study a growing number of genetic diseases—such as the Aarskog-Scott syndrome (Egorov and Polishchuk), a faciogenital dysplasia caused by mutations in a GEF protein (FGD1) regulating the Rho GTPase Cdc42–that are found to affect Golgi structure and function.

Altogether, topical reviews, hypothesis and theory articles, and original studies in this collection illustrate our recent progress in understanding Golgi biology, and also outline a specific set of yet unanswered Golgi-related questions. For example, exactly how do cargo and resident proteins travel to, through and out of the Golgi? What are the exact modes, carriers and molecular machineries of bi-directional Golgi trafficking? How are Golgi structure and its various functions modified during normal (differentiation, development, etc.) and abnormal (diseases, drugs, pathogens, etc.) circumstances?

## Author Contributions

KP, VL, YW, and JS prepared and wrote the manuscript.

### Conflict of Interest

The authors declare that the research was conducted in the absence of any commercial or financial relationships that could be construed as a potential conflict of interest.
